# Islands in the desert for cavity‐nesting bees and wasps: Ecology, patterns of diversity, and conservation at oases of Baja California Peninsula

**DOI:** 10.1002/ece3.5927

**Published:** 2019-12-17

**Authors:** Armando Falcón‐Brindis, María Luisa Jiménez Jiménez, Ricardo Rodríguez‐Estrella

**Affiliations:** ^1^ Conservation and Environmental Planning Program Centro de Investigaciones Biológicas del Noroeste (CIBNOR) La Paz México; ^2^ School of Natural Resources and the Environment University of Arizona Tucson Arizona

**Keywords:** bees and wasps, community ecology, oasis‐desert, pollinators, seasonality, trap nests

## Abstract

**Aims:**

The oases of Baja California Peninsula (BCP) have been proposed as important hotspots of biodiversity that hold an exceptional richness in the middle of desert conditions. We tested the effect of habitat and anthropogenic disturbance on communities of cavity‐nesting taxa, with specific emphasis on bees, wasps, and their natural enemies.

**Methods:**

In oases of BCP and desert neighbor environments, trap‐nesting taxa were evaluated in response to factors affecting the nest abundance, richness, and community structure. We used statistical models to find correlates of nest abundance and patterns of diversity, as well as ecological analyses to determine the effect of habitat and human disturbance on species diversity and community structure.

**Results:**

Solar irradiation, distance to a perennial waterbody and relative humidity influenced the presence of nests, number of brood cells, and parasitism. In general, abundance, species richness, and parasitism were higher in oases, especially in those with less human disturbance. Bees did not discriminate between oases and deserts to nest, whereas mud‐daubing wasps were more dependent of oases. The degree of anthropogenic disturbance did not affect the occurrence of parasitism, but it had an adverse effect on the parasitism intensity (number of attacked cells). The community structure was more complex and even in oases and low‐disturbed sites. The similarity between sites did not exceed 30%, and the proportion of shared species between oases and deserts varied from 2.7% to 26.6%.

**Main conclusions:**

The oases of Baja California are functioning as mesic islands in the desert, each oasis hosting a unique community of cavity‐nesting taxa. About 65% of the nests and 50% of species occurred exclusively in the oasis. Thus, cavity‐nesting species that depend on mesic conditions could be threatened if the oases of BCP disappear in the future. Local conditions in the oases and deserts of the BCP are shaping the community structure. However, large‐scale factors such as climate can influence the seasonality and occurrence of species within the community of cavity‐nesting dwellers. Since habitat loss and fragmentation can degrade the oases’ functionality, strategies to maintain the ecosystem services of pollination and biological control should be included in the conservation programs of these fragile habitats.

## Graphical abstract

Abundance and richness were higher in oases, about 65% of nest abundance and 50% of species occurred exclusively in the oasis. Bees did not discriminate between oasis and desert habitats to nest and mud‐daubing wasps were highly dependent of oases, thus at least 21 species could be threatened if the oases of BCP disappear in the future. Local conditions are shaping the community structure of species, but also large‐scale factors, for example, climate and biogeographic patterns seem to be influencing the community structure.

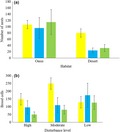

## 1 INTRODUCTION

Complex geological and paleoclimatic events have allowed the presence of oases in the desert of the Baja California Peninsula (BCP; Grismer, 2000). The oases from the BCP typically contain mesic vegetation (e.g., palms, common reed, cattails) and a permanent or semipermanent water body, which have created relictual habitats after a desertification process that began in the last glaciations by the late Pleistocene (Grismer & McGuire, 1993). Contrasting with the surrounding desert, the presence of water in oases allows the prevalence of a great biological and unique diversity (Grismer, 2000). However, these mesic habitats are considered vulnerable due to the small size, isolation, low connectivity, and especially because of continuous anthropogenic pressure (Arriaga & Rodríguez‐Estrella, 1997). The oases of BCP play a key role for the permanence and survival of species that require moist conditions (Rodríguez‐Estrella, 2004). They provide water and other resources inside arid environments where productivity is strongly regulated by extreme climatic events (Holmgren et al., 2006).

In oases of BCP, water has been crucial for the development of farming activities over the past 500 years, especially among oases with permanent waterbodies. This situation has provoked several human impacts (De Grenade & Nabhan, 2013). Moreover, the status of insect diversity in oases has been overlooked (Rodríguez‐Estrella, 2004). That is the case of keystone groups such as bees and wasps, which contribute in ecosystem functions through pollination (i.e., bees) and population control of small‐to‐moderate‐sized arthropods (i.e., wasps; Tscharntke, Gathmann, & Steffan‐Dewenter, 1998). Within these groups, the diversity of cavity‐nesting bees and wasps can positively respond to mesic habitats, which allow the prevalence of complex vegetation, thus offering a great amount of food and nesting resources (Flores, Zanette, & Araujo, 2018). In addition, both taxa are well known to respond to habitat structure (Loyola & Martins, 2008; Srba & Heneberg, 2012), patch size and connectivity (Holzschuh, Steffan‐Dewenter, & Tscharntke, 2010), and human disturbance (Collado, Sol, & Bartomeus, 2019; Gonçalves, Sydney, Oliveira, & Artmann, 2014). However, studies of cavity‐nesting bees and wasps are scarce in Mexico and have only focused on the nesting biology of few species (Domínguez & Jiménez, 2008; Rios‐Velasco et al., 2014).

Because of the importance of bees and wasps in ecosystem services and the relevance and threats in oases of BCP, an integrative approach through community ecology is urgently needed. In this work, we aimed to evaluate the diversity of cavity‐nesting taxa in oases of BCP and their surrounding desert area. We evaluated the occurrence (presence–absence) and abundance of nests and brood cells, parasitism, species richness, and community structure of cavity‐nesting taxa in oases and deserts under differential anthropic disturbance level. Assuming that cavity‐nesting bees and wasps respond to the complexity of habitat (Flores et al., 2018), we hypothesized that species diversity and community structure are shaped by the local habitat conditions, predicting that oases and more conserved habitats harbor higher species richness and less dominated communities (Balvanera et al., 2006; Gardener, 2014).

**Figure 1:**
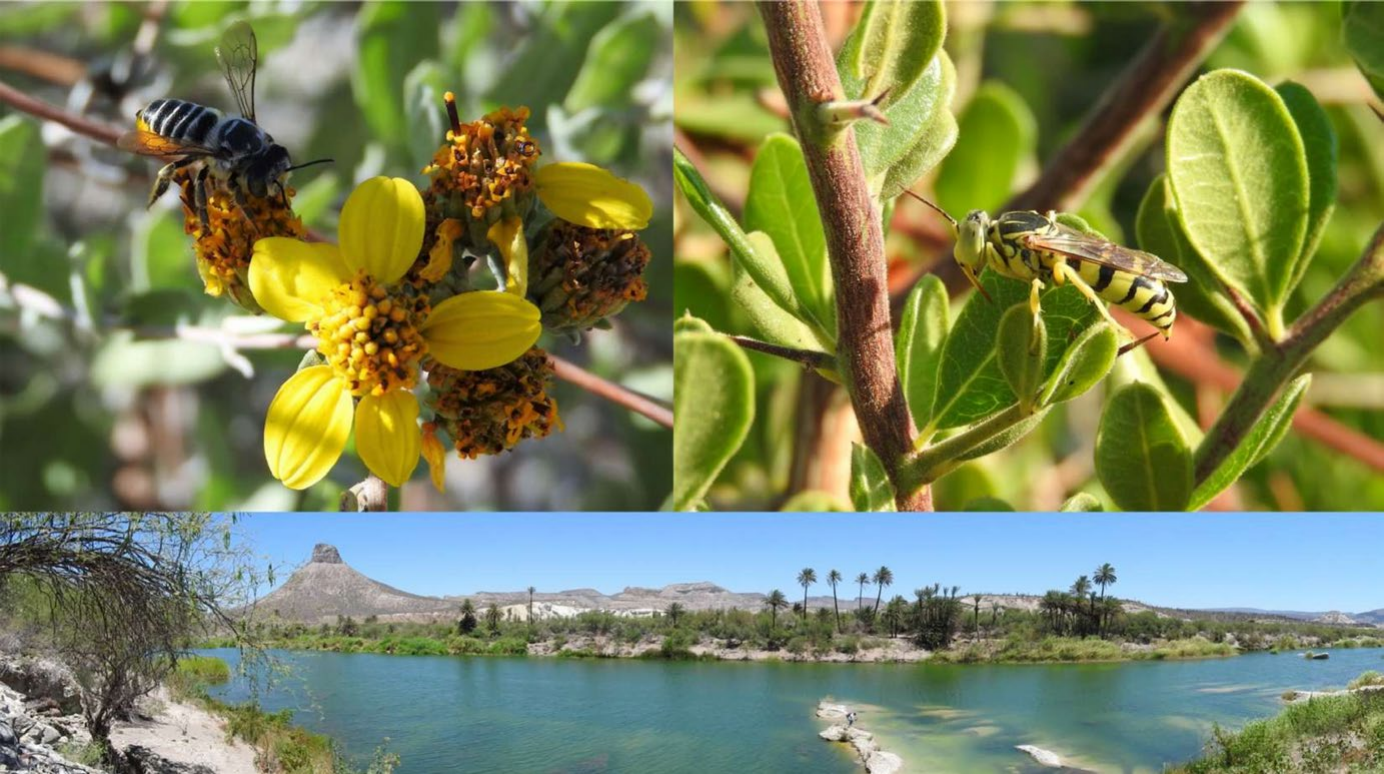
Bee and wasp species foraging on native plants at oasis in Baja California Peninsula. View of typical oasis in the desert

On the other hand, the geographic location and orientation (North to South) of the BCP allows an important climatic variation influencing on the patterns of species distribution and diversity (Morrone, 2005). Among other arthropods such as butterflies, scorpions, and ants, the patterns of diversity across the peninsula are variable (Brown, 1987; Due & Polis, 1986; Johnson & Ward, 2002). Surprisingly, although bees are well known to be more diverse in the xeric areas of the world (Michener, 1979), their distributional patterns across BCP remain unknown, especially since a great extension of the BCP is dominated by the Sonoran desert (Wiggins, 1980). Within this framework, we evaluated the effect of climatic variation in the BCP over the trap‐nesting communities as a complement to explain the variation in species composition and seasonality (Figure 1).

## 2 METHODS

### 2.1 Study area

**Figure 2:**
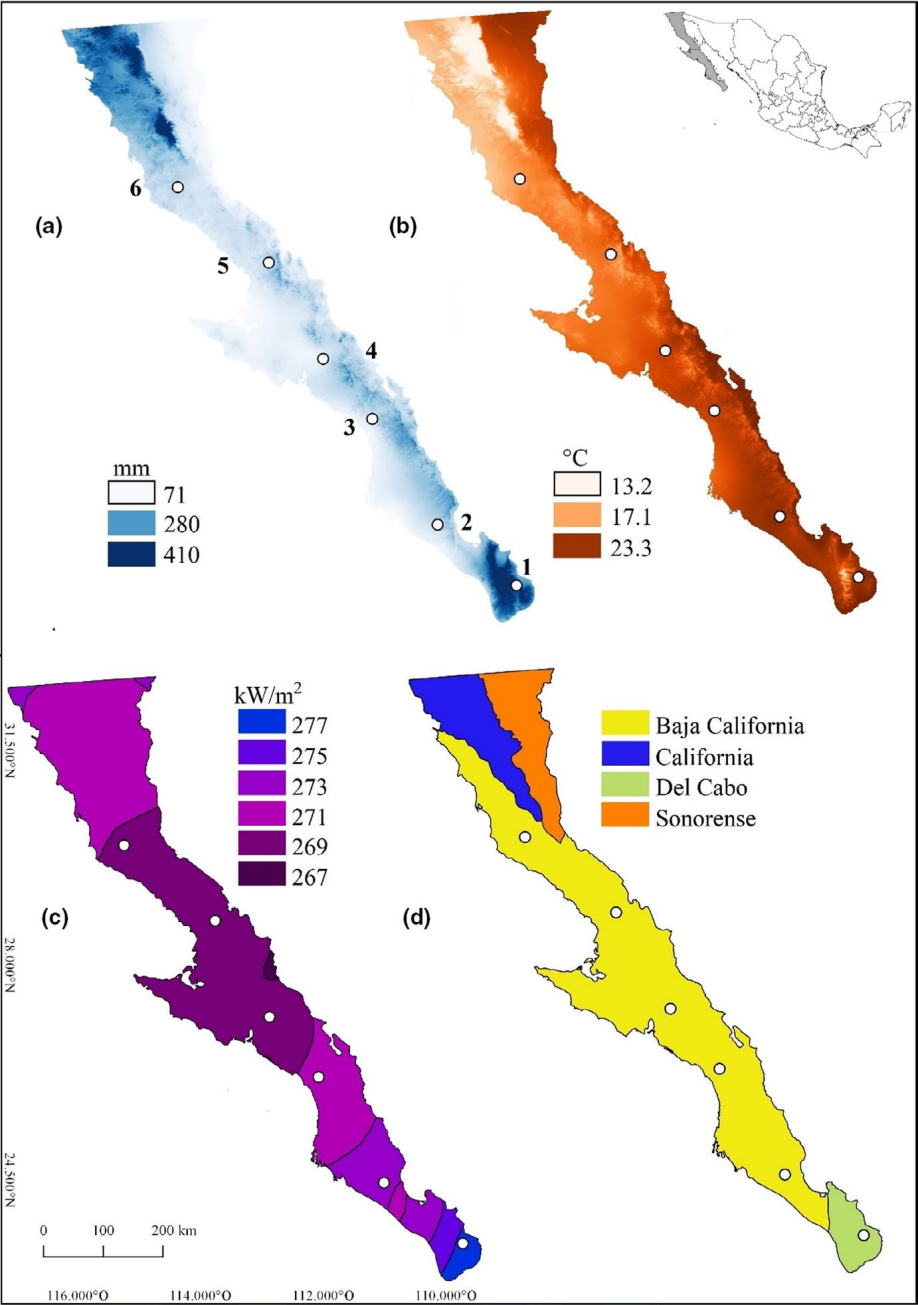
Studied localities in the peninsula of Baja California, Mexico. Sampling points are projected on maps with mean annual values of: (a) precipitation, (b) temperature, and (c) solar irradiation. Data of precipitation and temperature were obtained from WorldClim (Hijmans et al., 2005), and solar irradiation from the NOAA (DIVER, 2017). (d) biogeographic provinces (s*ensu* Arriaga et al., 1997). 1 = Santiago, 2 = El Pilar, 3 = La Purisima, 4 = El Sauzal, 5 = San Borja, 6 = San Fernando

Sampling was conducted in Baja California Peninsula (BCP), located in northwest Mexico. The north portion of the BCP (24°N to 32°N) belongs to the Nearctic region, which arid section is represented by xeric scrublands (hereafter treated as deserts), typical of the Sonoran desert (Wiggins, 1980). Within this region, the biogeographic provinces of Baja California, California, and Sonorense explain the distributional patterns of Nearctic organisms (Morrone, 2005). In the southernmost area (22°N to 24°N), the Del Cabo biogeographic province involves taxa with Neotropical affinity and contrasting vegetation such as tropical deciduous dry forest and oak–pine forest (Arriaga, Aguilar, Espinosa‐Organista, & Jiménez, 1997; Figure 2a–d).

There is a wet and dry season throughout the peninsula, with a rainfall period from July to September, ranging between 71 and 410 mm/year (Hijmans, Cameron, Parra, Jones, & Jarvis, 2005). Between 40% and 80% of the annual rainfall in the BCP is provoked by hurricanes and tropical cyclones, which frequency and intensity vary every year depending on large‐scale climate oscillations (e.g., El Nino and La Niña; Breña‐Naranjo, Pedrozo‐Acuña, Pozos‐Estrada, Jiménez‐López, & López‐López, 2015). In 2016, two tropical cyclones and one hurricane were recorded in the BCP, whereas in 2017, three tropical cyclones occurred in this area. However, during our samplings (2016–2017), the average rainfall followed a similar pattern of precipitation if compared with the last decade (DIVER, 2017; SMN, 2019). The heterogeneity across the BCP implies variation in geology, rainfall, solar irradiation, temperature, and humidity, which have shaped the biotic communities and distribution of species (Morrone, 2005). We selected six oases along a latitudinal range in the BCP. Each locality under its own climatic, geographical, and particular habitat conditions including the impacts by human activities (Table 1).

**Table 1:** Characterization of the oases

Oasis	Variables												
	Size (km^2^)	Lat	Long	Elev (m)	DNC (km)	Slope^a^	°C	mm	kW/m^2^	PW (km^2^)	Rh (%)	NA^b^	LHD
SA	1.47	23°28′	109°43′	132	17.4	Gulf	23.7	330.2	478.4	0.11	67.6	41	H
EP	0.25	24°28′	111°00′	120	27.3	Gulf	22.2	154.8	504.2	0.001	65.6	19	M
LP	2.69	26°11′	112°04′	95	20.9	Pacific	22.9	127	538.5	1.72	50.8	36	H
ES	0.21	27°10′	112°52′	150	53.3	Pacific	21.9	121.1	542.3	0.002	63.3	23	L
SB	0.06	28°44′	113°45′	445	27.9	Gulf	19.9	114.1	556.2	0.0005	42.1	16	M
SF	1.29	29°58′	115°14′	450	44.3	Pacific	18.2	91.1	562.2	0.0004	37.8	9	L

Note

Temperature (°C), precipitation (mm), and solar irradiation expressed as mean annual values (kW/m^2^) were obtained from SMN (2019). Geographical information was consulted on maps from INEGI (2016).

Abbreviations: DNC, Distance to nearest coast; EP, El Pilar; ES, El Sauzal; FP, Number of angiosperm species; H, high; L, low; LHD, Level of human disturbance; LP, La Purísima; M, moderate; PW, Perennial waterbody within the oasis; SA, Santiago; SB, San Borja; SF, San Fernando.

a Nearest slope.

b Includes both exotic and wild angiosperms in each oasis (HCIB and SDNHM Herbarium, 2018). LHD values were estimated in this study.

### 2.2 Experimental design

Our experimental unit was the oasis and its surrounding desert area, which we replicated along six sites through the time. Traps were set 1.5–2.0 m height and equally distributed in both oasis and desert areas to evaluate the fauna associated to each type of habitat. Since the oases are irregular patches of vegetation, we set the traps around the waterbodies, attempting to maintain a square shape of the trap distribution throughout the oases. We set the traps at one side of the oases and then set the same number of traps at the opposite direction. To evaluate the effect of water closeness on the reproductive fitness of bees and wasps (i.e., abundance of nests), we considered the largest perennial waterbody of each oasis as the focal point, then we set the traps in gradual distances (from around 5.0 m to 3,500 m) from the edge of the waterbody toward the desert area. We hypothesized that within a radius of 1,000 m around the oasis, wet conditions could still influence the abundance and richness of bees and wasps, and beyond this radius, we could find species adapted to xeric habitats.

**Figure 3:**
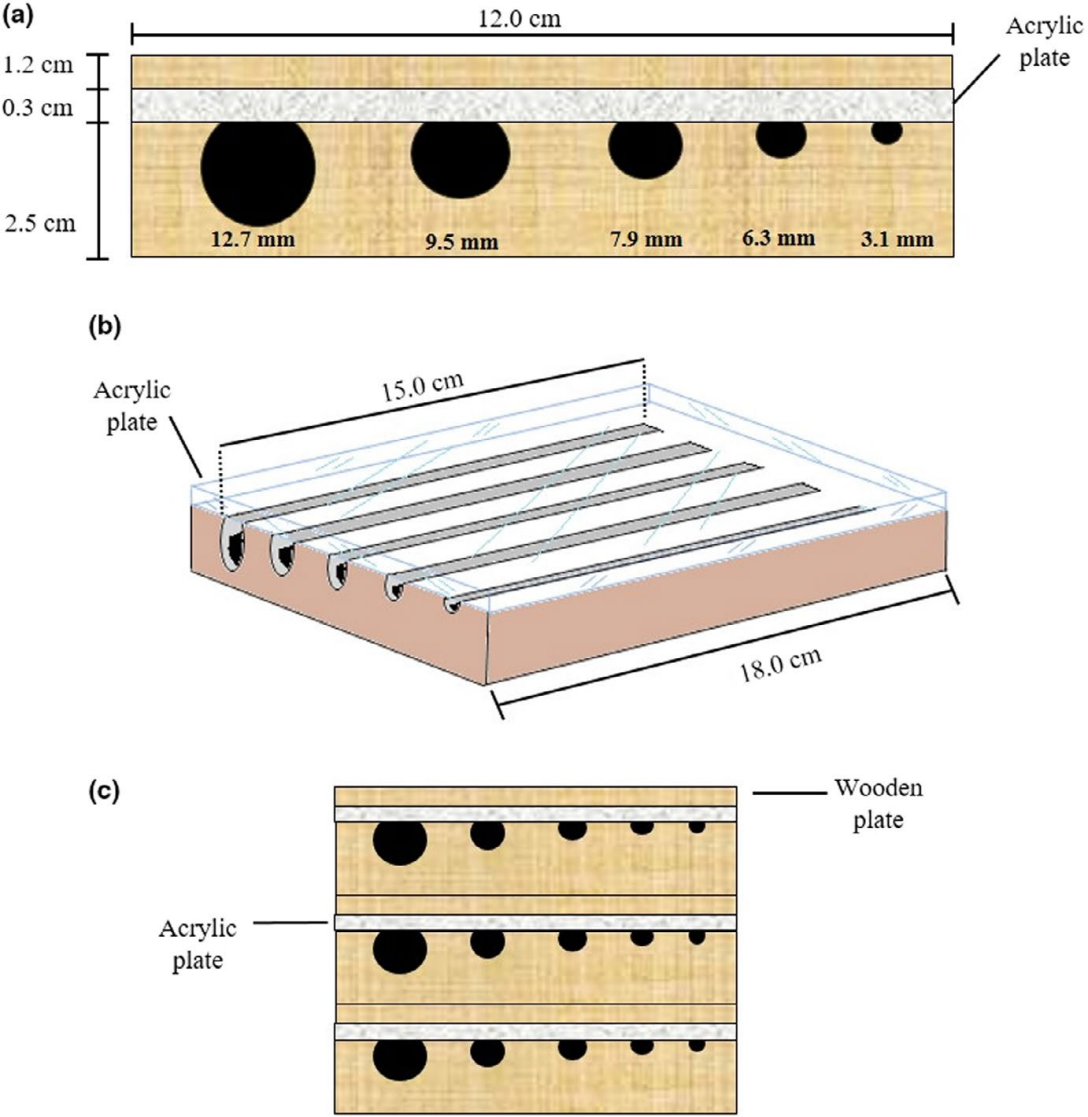
Internal and external design of trap nests. (a) Anterior and (b) upper lateral view. (c) Anterior view of three stacked traps

We set 30 trap nests at each locality. During the six months of sampling, we offered 5,400 cavities equally distributed in 180 trap nests set in the six localities. Trap nests consisted of three wooden blocks (12 × 18 × 2.5 cm each) stacked and gripped together, each with five rows of U‐shaped tunnels 15 cm long. Each row had different diameter (3.1, 6.3, 1.9, 9.5, and 12.7 mm) to increase the range of potential nesters and their fitness in mesic and xeric conditions (Krombein, 1967; Figure 3). These diameters were chosen according to the available commercial drill bits. From April to November 2016, we sampled the southernmost oases: Santiago (SA), El Pilar (EP), and La Purísima (LP). From April to October 2017, the northernmost oases were sampled: El Sauzal (ES), San Borja (SB), and San Fernando (SF). In both years, we replaced the occupied traps monthly. The nests were reared at room temperature (≈25°C) to evaluate cell content and emergence of natural enemies. Taxonomic identification was done in the laboratory of Arachnology and Entomology (CIBNOR), and at the ARS Bee Lab, UT.

### 2.3 Anthropogenic disturbance

To evaluate the effects of human activities on species diversity, we chose ten variables derived from human impacts: human population, population density, land‐use change, distance to the nearest settlement and paved road, intensity of agriculture, number of alien crops, slash‐and‐burn method, pesticide use and livestock (ranching; see Table S1 in the Appendix S1). We used a Canonical Correspondence Analysis to identify the variables explaining most of the anthropogenic disturbance at each locality. We used cluster analysis to classify the oases according to the similarity of human impacts (Johnson & Wichern, 2007). We incorporated these categories in the mixed models (as fixed effects) and diversity analyses. Multivariate analyses were done with *FactoMineR* package on R v3.6.1 (Le, Josse, & Husson, 2008; R Core Team, 2019).

In general, oases differed in the degree of human disturbance (*F*_5,54_ = 4.04, *p* = .0034). The values of human population, density, slash‐and‐burn, and land‐use change explained over 60% of the anthropogenic disturbance. With the cluster results, we categorized the six oases within three levels of disturbance: high = SA, LP; medium = EP, SB; low = ES, SF (Figures S1 and S2 in Appendix S1).

### 2.4 Data analysis

To determine the variables influencing the occurrence and abundance of nests and parasitism, as well as the species richness, we built generalized linear mixed models (GLMM). We fitted the GLMMs with Binomial and Poisson distribution for presence–absence and count data, respectively (Bates, Maechler, Bolker, & Walker, 2015). Binomial models were used to explain the occurrence of bee and wasp nests and parasitism, whereas Poisson models were built to analyze the abundance of nests, bee and wasp brood cells, parasitized cells and species richness. Since the number of brood cells per nest was not constant among bees and wasps, we evaluated the brood cell variation as a surrogate their fitness (i.e., potential offspring productivity), but also the shifts in the attacked cells from natural enemies (i.e., as a measure of the intensity of parasitism). We built separated models for bees, wasps, and species richness. The sampling locality was treated as the random effect (i.e., the grouping variable with six levels), and the fixed effects were those predictor variables (numeric and categorical) with specific effects over the nesting abundance (Bolker et al., 2009). The fixed effects were grouped into two major components: (a) Abiotic: solar irradiation, temperature, precipitation, and relative humidity; (b) Habitat: type of habitat (oasis or desert), distance of the trap to the largest perennial waterbody and human impacts (high, moderate, or low) calculated in this study (Table 1). These variables were the average monthly measurements from the last 20 years (SMN, 2019).

In order to avoid the overfitting in our models with unnecessary parameters, we tested the collinearity (i.e., high correlation between variables) of fixed effects. Collinearity was assessed by using three tools: pairwise scatter plots, Pearson correlation coefficients and the variance inflation factors (VIF). Pairwise plots helped to visualize the correlation between variables, then we selected those variables with less than *R* ^2^ = ±0.7 of correlation and VIF values less than three units (Zuur, Ieno, Walker, Saveliev, & Smith, 2009). The most parsimonious models were selected considering the second‐order Akaike information criterion (AICc; AIC for small samples), *p* values of goodness of fit tests, and explained deviance. We also verified that our models where not overdispersed (Bolker, 2015). The selection of the best minimum models is described in the Table S1 in Appendix S2. All models were fitted with *lme4* package (Bates et al., 2015) in R (R Core Team, 2019).

We used Cochran–Mantel–Haenszel test (*χ* ^2^_MH_) with repeated 2 × 2 tables, to evaluate if the abundance of bees and wasps between oases and deserts was equally distributed (Zar, 2010). We used the method of circle packing optimization as a hierarchical approach to understand the patterns of aggregation of bees and wasps in oases and deserts (Huang, Li, Li, & Xu, 2006). This approach selects the next circle to place according to the maximal hole degree rule. In this study, each circle represents the summarized occurrence of bees and wasps, following a hierarchical arrangement of distances from the waterbody. This method was calculated using the *packcircles* package (Bedward, Eppstein, & Menzel, 2018) in R (R Core Team, 2019).

### 2.5 Diversity analysis

For each locality, habitat, and disturbance level, we estimated species diversity of Hill numbers, order *q* = 0 (absolute richness) and *q* = 1 (the exponential of Shannon index for abundant species in the community) and dominance with Simpson index (*λ*; Chao, Chiu, & Jost, 2014). Differences in Shannon–Wiener diversity were estimated with the Hutcheson paired *t* tests for each group of habitat and human disturbance (Hutcheson, 1970). All of the former analysis was done with PAST (Hammer, Harper, & Ryan, 2001). Following the same approach of Hill numbers, the efficiency of sampling was analyzed through rarefaction curves for each locality and for both oases and deserts. The rarefaction curves were computed in R, using the *iNEXT* package (Hsieh, Ma, & Chao, 2019; R Core Team, 2019). We applied permutational analysis of variance (PERMANOVA) to test whether the type of habitat and level of human disturbance affect the community structure, then we tested homogeneity of dispersion (Anderson, 2001). The composition of species dissimilarity caused by habitat or disturbance was evaluated through nonmetric multidimensional scaling (NMDS) measured by Bray–Curtis distances (Kruskal & Wish, 1978). Both PERMANOVA and NMDS were done with the R package *vegan* (Oksanen et al., 2018; R Core Team, 2019). We used Sørensen quantitative Index to compare the similarities between oases and deserts using abundance data (Chao et al., 2014).

We used rank abundance models to evaluate the patterns of diversity. The rank abundance or dominance models (RAD) help to analyze the community distribution by plotting logarithmic abundance in response to species rank order (Whittaker, 1965). Several models have been proposed to represent the patterns of diversity (Magurran, 1988), but we followed Gardener (2014) to build models with our data. We built RAD models to evaluate the patterns of diversity in response to habitat and degree of human disturbance. The RAD models were fitted with Poisson distribution and selected by the lowest AIC value. We built the models with *vegan* package in R (Oksanen et al., 2018; R Core Team, 2019).

To analyze the beta diversity, the total dissimilarity (*β*_SOR_) was partitioned into two main components: nestedness (*β*_NES_) and turnover (*β*_SIM_). These components ranged from 0 to 1, where values close to 1 indicate more dissimilarity between sites (Baselga, 2010). Nestedness results when the community is the product of the original community after species loss caused by intentional external filters (e.g., human fragmentation). On the other hand, turnover arises by the replacement of species due to spatial or temporal limits. This approach is necessary for conservation efforts since high nestedness suggests a prioritization to preserve a small number of rich sites, while elevated turnover indicates that different sites should be preserved (Baselga, 2010). This analysis was computed using the *betapart* package (Baselga, Orme, Villeger, De Bortoli, & Leprieur, 2018) in R (R Core Team, 2019).

## 3 RESULTS

### 3.1 Nest occupation

**Figure 4:**
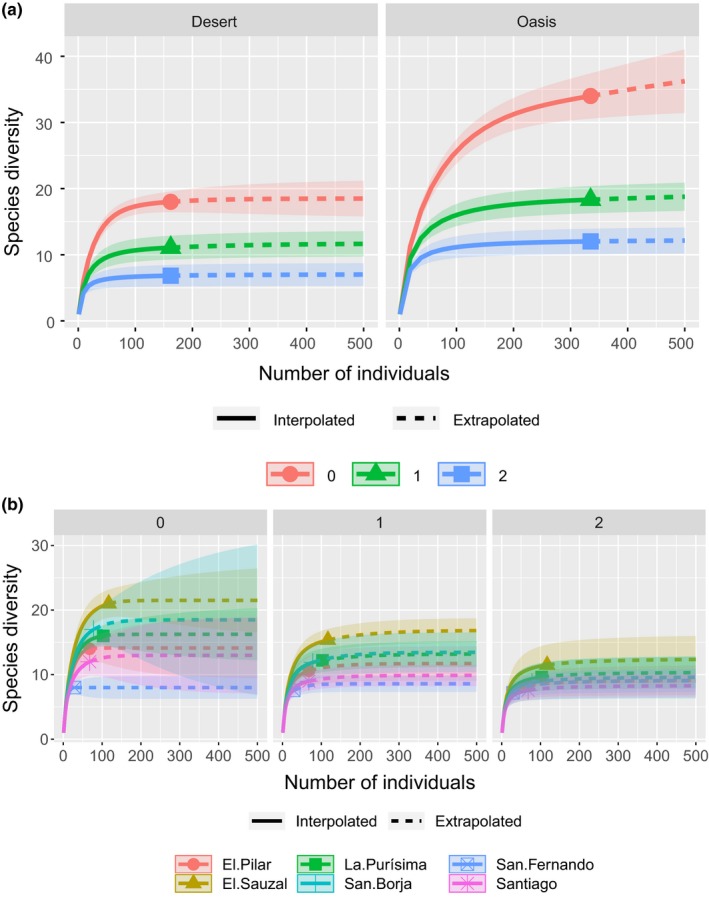
Species accumulation curves between (a) habitats and (b) sites. Hill numbers of order *q* = 0 (species richness), *q* = 1 (the exponential of Shannon entropy) and *q* = 2 (the inverse of Simpson concentration) were calculated for each measure and the respective 95% confidence interval

A total of 340 nests were occupied by 40 species. The pool of taxa included hymenopteran (33), dipteran (5), lepidopteran (1), and coleopteran orders (1). Within the nonparasitic bees, we found 12 species of six genera: *Euglossa* Friese (1 species), *Xylocopa* Latreille (2) (Apidae), *Hylaeus* Fabricius (1) (Colletidae), *Ashmeadiella* Cockerell (3), *Dianthidium* Cockerell (1), and *Megachile* Latreille (4) (Megachilidae). We found 13 species and nine genera of sphecoid and vespid wasps: *Trypoxylon* Latreille (3) (Crabronidae), *Stigmus* Panzer (1) (Pemphredonidae), *Auplopus* Spinola (1) (Pompilidae), *Isodontia* Patton (1), *Podium* Fabricius (1) (Sphecidae), *Euodynerus* Dalla Torre (1), *Leptochilus* Saussure (3), *Monobia* Saussure (1), *Parancistrocerus* Bequaert (1) (Eumeninae: Vespidae). The remaining 15 species were bees, wasps, dipterans, lepidopterans, and coleopteran parasites. The rarefaction curves indicated an appropriate sampling estimation between sites and habitats (Figure 4a,b).

### 3.2 Nesting dynamics

**Figure 5:**
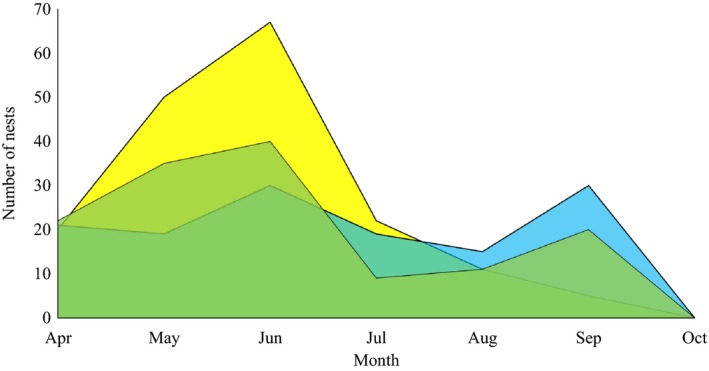
Average nest abundance of bees (yellow) and wasps (blue) through their active period. Green background represents the attacked nests from natural enemies

The peak of nesting activity varied along the peninsula and taxonomical group. In the three southernmost localities (23° to 26°N), the peak of abundance was in May; in the three northernmost sites (27° to 30°N), the maximum abundance was in June. The nesting activity fully stopped in October. However, in the northernmost site (i.e., SF) the activity occurred only from May to August. While the peak abundance occurred during the dry season for both bees and wasps, the two groups did not follow the same temporal pattern. While bee abundance falls after the peak of nesting activity in the late spring, the activity of wasps had two peaks, in spring and fall. Natural enemies primarily targeted wasp species. The highest rate of attacks from natural enemies occurred in the mid‐spring (80.2%) and fall (19.8%), which was significantly correlated with the peak of bee activity (*r* = .84, *p = *.017) and less strong and nonsignificantly with wasps (*r* = .53, *p = *.21; Figure 5).

### 3.3 Responses to habitat and disturbance

According to the GLMMs, the overall occurrence of nests, the number of brood cells and the presence and intensity of parasitism, were positively influenced by the solar irradiation, relative humidity, and the distance to the perennial waterbody in the oasis. In general, the effect of these variables was weak if compared to the strong negative effect of the habitat type over the presence of wasp nests and number of wasp's brood cells (see the estimate values of each model in Table 2). The habitat type did not affect the presence of bee nests nor the amount of their brood cells. In addition, the level of human disturbance showed a strong negative effect over the number of brood cells into the nests of bees and wasps, but also in the number of parasitized cells (Table 2).

**Table 2:** Best GLMMs explaining the presence nests, parasitism and abundance of brood cells

Variable	Presence of bee nests^a^				Presence of wasp nests^a^			
	Estimate	*SE*	*df*	*p*‐Value	Estimate	*SE*	*df*	*p*‐Value
Solar irradiation	0.272	0.005	1	**<.001**	0.258	0.006	1	**<.001**
Relative humidity	0.74	0.017	1	**<.001**	0.093	0.024	1	**<.001**
Distance to water	0.477	0.002	1	**.024**	0.001	0.004	1	**<.001**
Habitat	−0.251	0.37	1	.545	−3.19	0.53	1	**<.001**

	Number of bee brood cells^b^				Number of wasp brood cells^b^			
	Estimate	*SE*	*df*	*p*‐Value	Estimate	*SE*	*df*	*p*‐Value
Disturbance	−1.12	0.219	1	**<.001**	−1.074	0.237	1	**<.001**
Solar irradiation	0.002	0.001	1	**<.001**	0.016	0.004	1	**<.001**
Relative humidity	0.071	0.008	1	**<.001**	0.105	0.012	1	**<.001**
Distance to water	0.004	0.005	1	**<.001**	0.001	0.0001	1	**<.001**
Habitat	0.202	0.111	1	.069	−1.940	0.166	1	**<.001**

	Presence of parasitized nests^a^				Number of parasitized cells^b^			
	Estimate	*SE*	*df*	*p*‐Value	Estimate	*SE*	*df*	*p*‐Value
Disturbance	−1.197	1.22	1	.326	−4.283	3.506	1	**<.001**
Solar irradiation	0.064	0.011	1	**<.001**	0.058	0.004	1	**<.001**
Relative humidity	0.186	0.038	1	**<.001**	0.175	0.016	1	**<.001**
Distance to water	0.001	0.0004	1	**<.001**	0.0005	0.001	1	**<.001**
Habitat	−2.012	0.544	1	**<.001**	−0.766	0.171	1	**<.001**

Note

Binomial models account for the presence of bee and wasp nests and parasitism, whereas Poisson models represent the counts of individual brood cells and parasitized cells. Habitat (oasis or desert), Distance to water = Distance from nest to nearest perennial waterbody inside the oasis. Values of *p* < .05 are shown in bold. The estimate values indicate the direction of the relationship.

Abbreviations: *df*, degrees of freedom; *SE*, standard error.

a Binomial models (presence–absence).

b Poisson models (counts).

**Figure 6:**
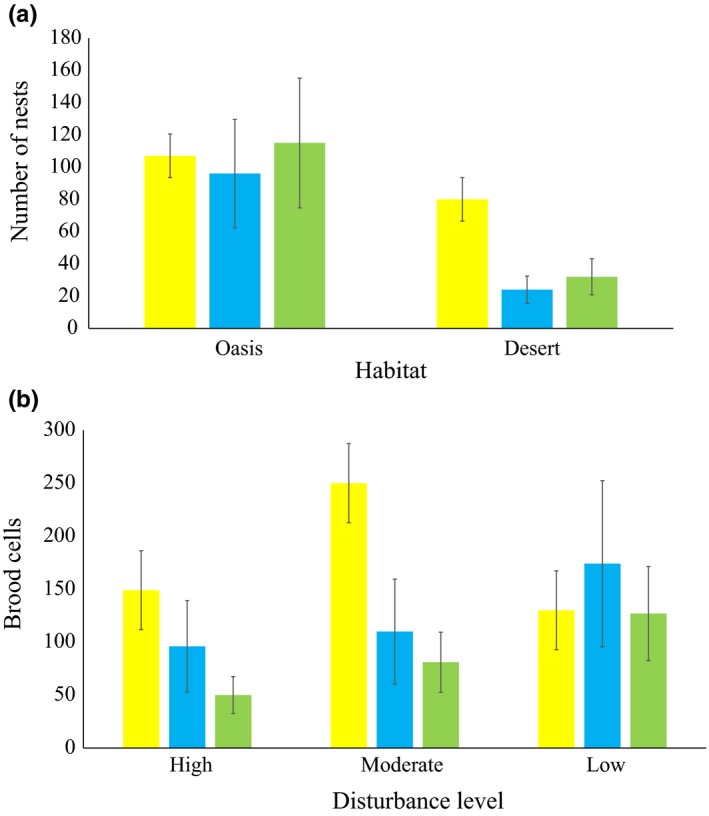
(a) Nest abundance of cavity nesters between habitat types. (b) Brood cell abundance of cavity nesters along the gradient of human disturbance. Bees (yellow bar), wasps (blue), and natural enemies (green). Data are *x* + standard error

Localities had different proportions of bee and wasp nests in oases and deserts (*χ* ^2^_MH_ = 5.13, *df* = 1, *p = *.023). No nests occurred in the xeric area of Santiago. The overall proportion of nests and attacks from parasites was higher in oases (*χ* ^2^_1_ = 18.7, *df* = 1, *p < *.01; Figure 6a). However, bees did not discriminate between oases and deserts to nest (*χ* ^2^_1_ = 0.028, *p = *.866), whereas wasps were more abundant in oases (*χ* ^2^_1_ = 38.5, *p < *.01). In high‐disturbed sites, we found the largest proportion of bee and wasp nests (*G* = 6.15, *df* = 2, *p* = .04), but the least amount of attacked brood cells by natural enemies (*χ* ^2^ = 42.3, *df* = 1, *p < *.001; Figure 6b).

**Figure 7:**
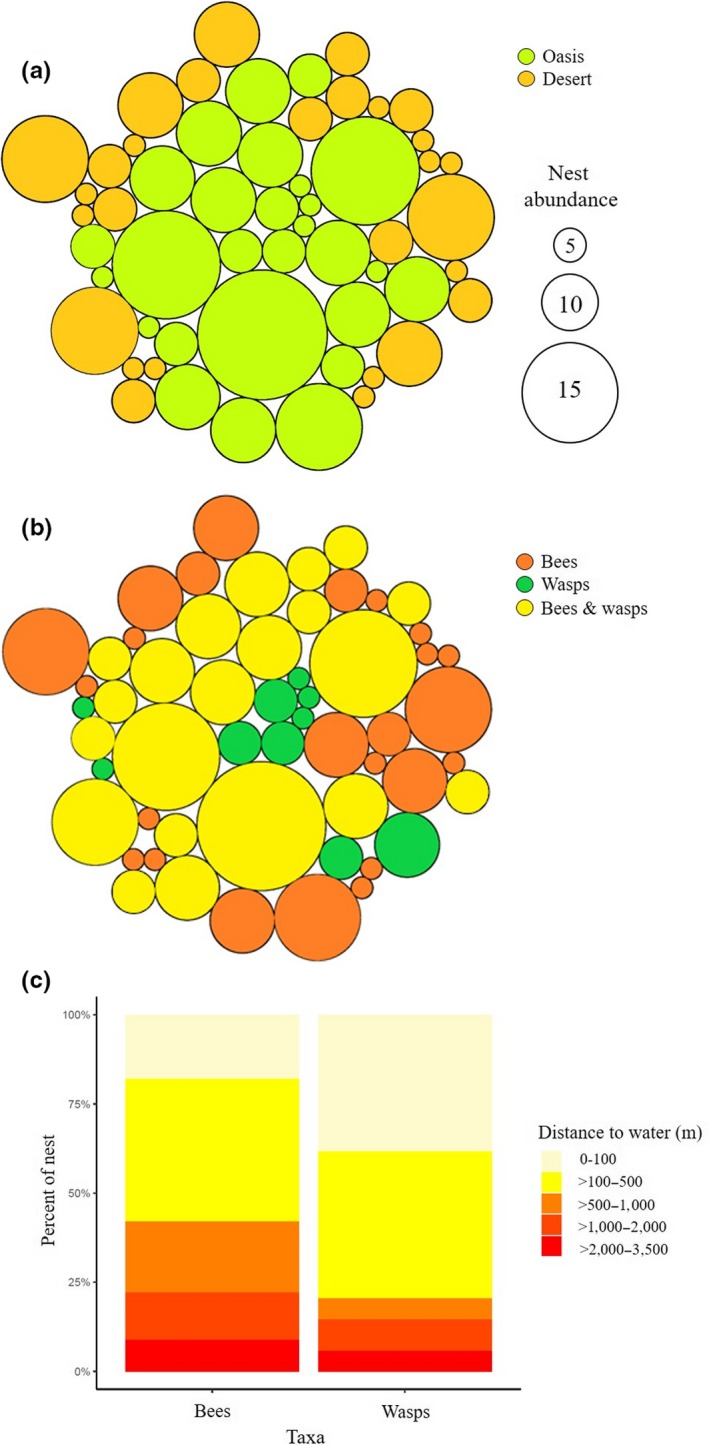
Simplified spatial representation of hymenopteran nest aggregation around the waterbody in (a) oases and deserts and (b) bees and wasps. (c) Rank of distances of nest occurrence. Circles have a concentric and hierarchical arrangement of both abundance and distance to waterbody. Each circle represents the summarized occurrence of bees and wasps at different distances from the waterbody. The diameter indicates the total abundance at each observation. For this representation, all oases and deserts were considered

In general, the 64.8% of all nests occurred in oases, within a radius of 350 m from the perennial source of water. The 73% of wasp nests were found at 0 m–500 m from the waterbody's edge, whereas the 72% of bee nests occurred at 0 m–1,000 m. Both bees and wasps co‐occurred at 100 m–500 m from the waterbody, but particularly the group of mud‐daubing wasps nested within a radius of 100 m around the waterbody and vespid wasps occurred more frequently in the desert habitats (Figure 7a–c).

Out of the total 40 species, 21 only occurred in oases, seven in xeric environments (deserts), and the remaining 12 in both habitats. Species within the group of spider‐hunting (genus *Trypoxylon*) and spheciform (*Podium*, *Stigmus*) wasps, leaf‐cutting bees (*Megachile*), and an orchid bee species (*Euglossa*) were restricted to the oases. However, there were species from the same genus (excepting *Euglossa*) that nested either in oases or in deserts. The 89% of caterpillar‐hunting wasps (Eumeninae) occurred in the desert.

### 3.4 Effects on species diversity

With the GLMMs, we found that the species richness of bees and wasps is negatively affected by the human disturbance. Climatic variables such as the solar irradiation, relative humidity, precipitation, and temperature showed a weak but significant effect over the species richness (Table 3).

**Table 3:** Best GLMMs explaining the bee and wasp richness

	Bee richness				Wasp richness			
	Estimate	*SE*	*df*	*p*‐Value	Estimate	*SE*	*df*	*p*‐Value
Disturbance	−0.743	0.224	1	**<.001**	−1.144	0.345	1	**<.001**
Solar irradiation	0.008	0.002	1	**.003**	0.001	0.001	1	.329
Relative humidity	0.03	0.008	1	**<.001**	−0.025	0.0001	1	.082
Precipitation	−0.028	0.007	1	**<.001**	−0.0002	0.003	1	.946
Temperature	−0.018	0.036	1	.609	−0.081	0.04	1	**.045**

Note

Models were fitted with Poisson distribution of error.

**Figure 8:**
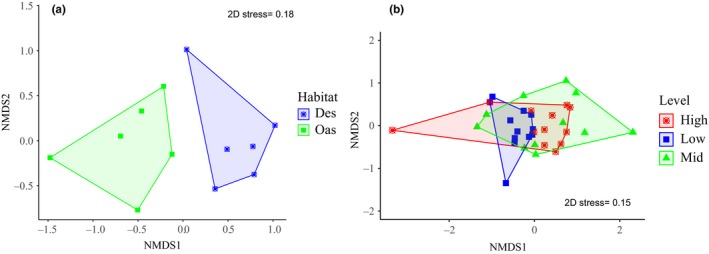
Community composition shaped by (a) habitat type and (b) level of human disturbance using Nonmetric multidimensional scaling (NMDS). Des = desert, Oas = oasis

**Figure 9:**
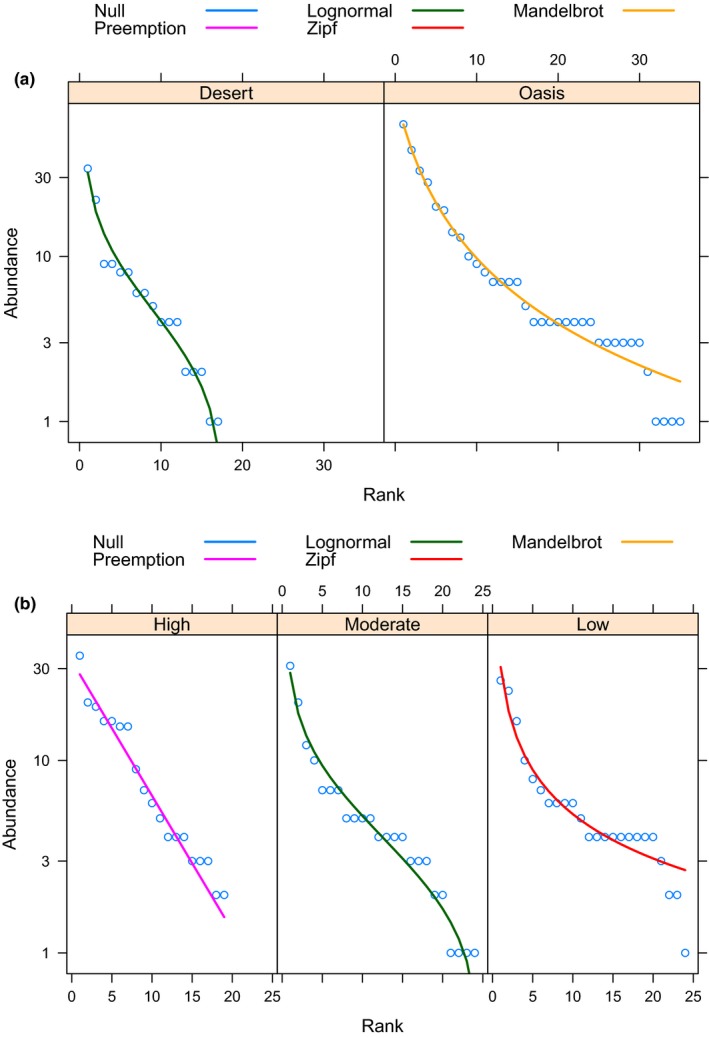
Rank abundance or dominance models (RAD). The RAD models represent the community structure in response to (a) habitat and (b) level of human disturbance. Follow Gardener (2014) for detailed explanation of each fitted model. The community structure in the desert was less even than the oasis, and the complexity of the community was inverse to the level of human disturbance

The species richness varied with habitat, disturbance level, and locality (Table 4). The highest number of species and lowest levels of dominance (*λ*) were found in oases with low level of disturbance. Differences in Shannon diversity were found between oasis and desert (*t* = 4.95, *df* = 324, *p *< .001) and between low and highly disturbed sites (*t* = 4.12, *df* = 316.8, *p* < .001). The community structure of hymenopterans was significantly affected by both habitat type (PERMANOVA: *F* = 3.4, *df* = 1, *p = *.007) and level of human disturbance (*F = *2.7, *df* = 2, *p* = .001; Figure 8a,b). The RAD models fitted different patterns of diversity according to the habitat and level of disturbance. Compared with the desert, the community of an oasis remains more complex and less dominated. Likewise, the sites with low and moderate disturbance presented less dominated communities. In contrast, the community of highly disturbed conditions showed the least evenness values, fitting within a curve of geometric series pattern (Figure 9a,b).

**Table 4:** Total species richness and abundance of bees, wasps and natural enemies among habitats, anthropogenic disturbance, and locality

		*N*	*S*_bees_	*S*_wasps_	*S*_nat.enem._	*S*_total_	Q1	*λ*
Habitat	Oasis	219	8	11	14	33	18.1	0.084
	Xeric desert	121	8	5	5	18	10.4	0.158
Disturbance	Low	108	7	8	9	24	17.9	0.075
	Medium	112	7	7	10	24	15.8	0.090
	High	120	6	7	6	19	13.7	0.091
Locality	SA	51	4	3	5	12	10.14	0.109
	EP	49	5	3	6	14	10.71	0.118
	LP	73	4	6	6	16	12.33	0.102
	ES	87	5	7	9	21	15.05	0.087
	SB	52	6	4	7	17	11.89	0.114
	SF	28	4	3	1	8	7.55	0.139

Note

*N = *Abundance of individuals, *S = *richness, *λ* = Simpson's dominance, Q1 = Hill number of first order.

**Figure 10:**
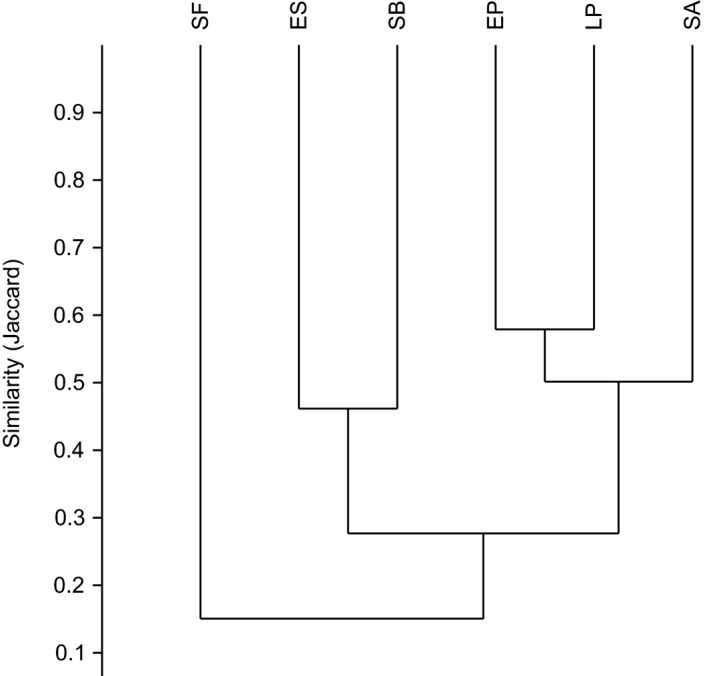
Overall similarity of species between the studied sites. The dendrogram is measured with Jaccard similarity. ES, El Sauzal; LP, La Purísima; SA, Santiago; SB, San Borja; SF, San Fernando

Most pairwise comparisons between oases and deserts showed differences in species diversity (Table 5). The Sørensen quantitative Index (*C*_N_) indicated 27.3% of overall similarity between oases and xeric deserts and was equally low between both habitats (*t* = 1.15, *df* = 14, *p* = .26; Table 5). While five of the six sites shared 30% of species (*I*_J_ = 0.30), the northernmost locality of San Fernando (30°N) had the lowest value of similarity (*I*_J_ = 0.17; Figure 10). The similarity between oases and deserts within localities was also low, ranging from 2.7% to 26.6%.

**Table 5:** Pairwise comparisons of diversity and similarity between localities and their oases and deserts

Localities	*H*′ diversity			*C*_N_	
	*t* Value	*df*	*p*	Oasis	Desert
SA‐LP	−2.65	150.8	.0089	0.50	0.05
SA‐ES	−4.57	159	<.001	0.42	0.06
SA‐SB	−2.09	145.2	.0376	0.28	0.05
EP‐ES	−2.99	147.5	.0032	0.44	0.04
EP‐SB	−0.83	145.8	.4038	0.22	0.25
EP‐SF	2.55	87.1	.0125	0.08	0.20
LP‐ES	−2.11	219.7	.0355	0.54	0.26
LP‐SF	4.11	75.3	<.001	0.04	0.28
ES‐SB	2.00	159.9	.047	0.45	0.44
ES‐SF	6.01	79.2	<.001	0.08	0.19
SB‐SF	3.36	94.4	.0012	0.06	0.25

Note

*H′* = Shannon–Wiener, *C*_N_ = Sørensen quantitative Index, *df* = degrees of freedom. Only comparisons with *p* < .05 are shown.

Abbreviations: EP, El Pilar; ES, El Sauzal; LP, La Purísima; SA, Santiago; SB, San Borja; SF, San Fernando.

A high dissimilarity of species between sites (*β*_SOR_ = 0.70) and habitats (*β*_SOR_ = 0.86) was found according to beta diversity analysis. Species turnover was the main component of dissimilarity in both sites (*β*_SIM_ = 0.59, *β*_NES_ = 0.11) and habitats (*β*_SIM_ = 0.78, *β*_NES_ = 0.07).

## 4 DISCUSSION

### 4.1 Seasonality

The climatic variables affecting the phenology of both groups were the solar irradiation, relative humidity, and precipitation. The seasonality of insects tends to increase with latitude, as the active period depends on suitable climatic conditions, usually present within spring and summer in temperate regions or even shorter at upper latitudes (Wolda, 1988). This may explain why the peak of nesting activity varied between the southern and northern sites, and why the locality at the highest latitude (i.e., SF, 30°N) had the shortest span of activity (May to August). This oasis is located in the limits of the biogeographic provinces of Baja California and California, the latter with lower temperatures and with winter rainfall typical of Mediterranean climates (Morrone, 2005).

Much of the seasonality can be explained by the spatiotemporal patterns of solar irradiation along the BCP, since the fitness of most arthropods is more effective in environments with higher levels of solar irradiation (Herrera, 1995, 1997). However, other climatic variables such as precipitation, humidity, and temperature may be playing a role in the nesting activity. For instance, the null occurrence of nests in the desert area of Santiago, which is the hottest locality (23.7°C of average annual and extremes of 44°C during May–July) that receives the least amount of solar irradiation might be an indicator that such temperatures may be exceeding the thermal threshold of cavity‐nesting dwellers (Terblanche, 2013). In fact, Santiago has been previously highlighted because of its distinctive arid and hot conditions within the Cape region (Díaz & Troyo, 1997).

At the regional scale, both climatic and geographic heterogeneity of the BCP may have complex effects on the seasonality of these insects (Wolda, 1988). Moreover, shifts in rainfall promoting hot and moist conditions (El Niño) or cool and dry weather (La Niña) in the northern hemisphere may induce differential responses in the ecosystems of the BCP (Caso, González‐Abraham, & Ezcurra, 2007). During 2015 and 2016, there was a strong El Niño influence over the tropical Pacific. In contrast, 2017 had a weak influence of La Niña (NOAA, 2019). These climatic oscillations may have influenced the seasonality of bees and wasps in this study. Nonetheless, the oases of the BCP may be acting as mesic refugia for several species in order to tolerate extreme climatic phenomena that can shape arid and semiarid ecosystems (Holmgren et al., 2006).

### 4.2 Habitat and disturbance effects

We found that the type of habitat and level of human disturbance influenced the diversity, community structure, and parasitism. The diversity and parasitism were higher in oases and low‐disturbed sites. Moreover, the RAD models indicated that communities were more even and complex in oases and preserved sites. Human‐altered habitats can host an important number of bee species, and usually a greater abundance if compared with preserved habitats (Collado et al., 2019). However, habitat loss and fragmentation can negatively affect the richness of trap‐nesting taxa and the interactions with their parasites and simplify the community assemblage (Cane, Minckley, Kervin, Roulston, & Williams, 2006; Carrié et al., 2017; Ferreira et al., 2015; Holzschuh et al., 2010; Schüepp, Rittiner, & Entling, 2012; Staab, Pufal, Tscharntke, & Klein, 2018). Similarly, our results support the hypothesis that human‐altered habitats have adverse effects in the community structure and species richness. Anthropogenic disturbance was also a negative consequence for the host‐parasite interactions, reducing the intensity of attacked cells per nest in highly disturbed sites. In this sense, it is largely known that habitat simplification affects higher trophic levels (i.e., parasites) as richness and abundance of lower trophic levels (i.e., host species) decrease (Araujo, Fagundes, & Antonini, 2017; Flores et al., 2018; Steffan‐Dewenter & Schiele, 2008; Tscharntke et al., 1998).

We found that the overall rate of parasitism varied from 20% to 100% of brood cells per nest. The attacks from nest parasites mainly targeted mud‐daubing wasp species in the oases, but also bee species such as *M. occidentalis* and *M. gentilis* were frequently parasitized. We found nests of *M. occidentalis* entirely parasitized by bombilid flies and those nests of *M. gentilis* heavily attacked by eulophid wasps. However, it has been proposed that the attack rate, and diversity of parasitic species depend on the season, richness and abundance of the host species and habitat conditions (Krombein, 1967; Schüepp, Hermann, Herzog, & Schmidt‐Entling, 2011; Tscharntke et al., 1998). In our results, the type of habitat also influenced the intensity of parasitism, being higher in oases than in deserts.

On the other hand, since trap‐nesting hymenopterans are spatially limited by available pre‐existing cavities, food, and physiological fly restrictions, local habitat configuration become crucial (Gathmann & Tscharntke, 2002; Loyola & Martins, 2011; Zurbuchen et al., 2010). This may partially support the striking contrast in abundance and species composition between oases and desert habitats, indicating that local conditions are playing a major role in the structure of their communities. However, the differential responses between bees and wasps could be due to distinct spatiotemporal patterns in habitat preferences, floral and prey resources, and life history (Gonçalves et al., 2014; Tylianakis, Klein, & Tscharntke, 2005).

The strong dependence of water is usual in mud‐daubing wasps, since they require a near source of mud to build up their nests (Morato & Martins, 2006). Similarly, nests of *Trypoxylon*, *Auplopus*, *Podium* wasps occurred within 100 m from the water source. However, other mud‐user wasps (*Euodynerus*, *Monobia*, *Leptochilus*, *Parancistrocerus*, *Trypoxylon tridentatum*) were not confined to oases. These species have different life histories, nesting and hunting strategies to survive in dry conditions (Krombein, 1967). For instance, *T. tridentatum* is highly adapted to hunt and nest in the xeric scrublands of Arizona (Krombein, 1967) and southern BCP (Domínguez & Jiménez, 2008). Moreover, we believe that the presence of ephemeral ponds formed during the rainy season in the desert may serve as temporary mud sources for some wasps (Whitford, 2002). In contrast, the presence of water did not seem to be a restrictive resource for bee nesting, since the bee species utilizing the traps were not mud dependent. Nonetheless, the megachilid species had different habitat preferences. While the resin user *M. occidentalis* was more abundant in desert habitats, leaf‐cutting species such as *M. gentilis* and *M. inscita* were common in the oases. It suggests that probably their occurrence depend on the presence of suitable substrate (e.g., leaves, resins) or preferred pollen sources (Armbrust, 2004).

### 4.3 Large‐scale effects

At a large scale, climate conditions may have a major influence on the species richness and composition in the oases of BCP, as it has been observed in most North–South orientated peninsulas (Battisti, 2014; Gaucherel, Tramier, Devictor, Svenning, & Hély, 2018; Taylor & Regal, 1978). Moreover, patterns of species diversity along the BCP are largely influenced by paleoclimatic and paleogeographic events affecting differently both vertebrate (Álvarez‐Castañeda & Murphy, 2014; Gonzalez‐Rubio, García‐De León, & Rodríguez‐Estrella, 2016; González‐Trujillo et al., 2016; Graham, Bryson, & Riddle, 2014) and invertebrate taxa (Brown, 1987; Due & Polis, 1986; Johnson & Ward, 2002). In this sense, climate variation should be an important factor to consider when interpreting ecological data across the BCP. Such factor may help to understand the patterns of diversity found throughout the sampled sites, especially the distinctive species composition at the extreme hot tropical (23°N) and cold arid (30°N) localities. The influence of the Nearctic and Neotropical regions throughout the BCP may be playing a role in the delimitation of resources or physiological boundaries of species (Morrone, 2005). For example, the occurrence of *Megachile inscita* or the orchid bee *Euglossa viridissima* exclusively in the southernmost tropical oasis (Falcón‐Brindis, Ayala, Jiménez, & Hinojosa‐Díaz, 2018), and the presence of Nearctic species such as *Dianthidium parvum* or *A. curriei* only at the northernmost oases.

On the other hand, an intrinsic low similarity of arthropod species among oases (Jiménez & Palacios‐Cardiel, 2010; Jiménez, Palacios‐Cardiel, & Tejas, 1997) may be obscuring large‐scale patterns of diversity. In this work, we found a generalized low number of shared species between habitats and sites. Excepting by the northernmost locality, the similarity seemed to be influenced by the distance between sites. It has been suggested that the low similarity in oases is the result of ecological isolation processes produced by large‐scale historical events (e.g., glaciations, vicariant oscillations, and desertification; Grismer, 2000). In this regard, each oasis should be seen as the product of unique combined natural (biotic and abiotic) and synthetic (anthropogenic) processes continuously shaping the diversity.

### 4.4 Implications for conservation

Since preserved natural areas can maintain the diversity of pollinators (Collado et al., 2019), it is urgently required to evaluate the status of pollinators especially in critical isolated habitats (Ghazoul, 2015). It has been shown that mesic habitats can be important hotspots of bee and wasp diversity in semiarid conditions (Flores et al., 2018). In this work, we present strong evidence supporting the unique species composition of oases and their importance as critical (mesic) habitats within xeric ecosystems. Besides, the high species turnover suggests that these oases should be preserved, since they host exclusive communities of trap nesters (Baselga, 2010).

Land‐use change is one of the most important factors causing habitat loss and the reduction in bee and wasp diversity (Holzschuh et al., 2010). The decrease of plant richness and nesting resources are among the negative consequences of land‐use change and may have direct implications on these hymenopterans (Schüepp et al., 2011). In this regard, we found that human alteration modifies the community structure of bees and wasps and reduces the interactions with their natural enemies. Alien plants can also disrupt the interactions between native assemblages of pollinator angiosperms (Stout & Morales, 2009). However, this process may vary according to the plant taxa and their capability of dispersion (Vilà & Weiner, 2004). In oases of Baja California Peninsula, invasive plants have been documented to be a serious problem threatening native vertebrate and invertebrate species that depend on these habitats (Rodríguez‐Estrella, Pérez‐Navarro, Granados, & Rivera, 2010).

Our results showed that the oases of Baja California Peninsula are acting as mesic islands in the middle of desert conditions, supporting a great abundance, diversity, and unique assemblages of cavity‐nesting bees, wasps, and their natural enemies. The dependence to these habitats was significant for mud‐daubing wasps, but more species, including other bees and wasps should be depending on the resources provided only by oases (e.g., water, exclusive food, and nesting resources). In addition, the oases and low‐disturbed sites sustained complex communities of cavity‐nesting taxa. However, these habitats are under continuous anthropogenic pressure threatening the ecological balance for these keystone insects. Therefore, conservation of these critical habitats should consider maintaining the functional roles of bees and wasps at the oases of BCP.

## CONFLICT OF INTEREST

None declared.

## Supporting information

AppendixS1‐S2

## Data Availability

The data used for this research are deposited in Dryad, including species list and biodiversity data. https://doi.org/10.5061/dryad.ghx3ffbjq.
