# Supplementary material for: Islands in the desert for cavity‐nesting bees and wasps: Ecology, patterns of diversity, and conservation at oases of Baja California Peninsula
Source: Ecol Evol. 2019 Dec 17;10(1):527–42. doi: 10.1002/ece3.5927 (PMC6972840; doi:10.1002/ece3.5927)
Supplement: Supplementary file 1 — AppendixS1‐S2 [file ECE3-10-527-s001.docx]

Ecology and Evolution

**SUPPORTING INFORMATION**

**Islands in the desert for cavity-nesting bees and wasps: ecology, patterns of diversity, and conservation at oases of Baja California peninsula**

Armando Falcón-Brindis, María Luisa Jiménez Jiménez & Ricardo Rodríguez-Estrella

**Appendix S1.** Characterization of oases according to the degree of human impacts.

**Table S1.** Anthropogenic variables associated to human impacts. Variables were transformed and weighted for multidimensional analyses. SA=Santiago, EP=El Pilar, LP= La Purísima, ES=El Sauzal, SB=San Borja, SF=San Fernando. Variables were standardized and then weighted considering the land-use change as the highest impact variable (following Mantorell & Peters, 2005).

| Variable | SA | EP | LP | ES | SB | SF |
| --- | --- | --- | --- | --- | --- | --- |
| Human population | 800 | 15 | 500 | 10 | 10 | 0 |
| Population density (ind/ha) | 5.4 | 1 | 1.9 | 0.5 | 1.6 | 0 |
| Land-use change (%) | 90 | 65 | 60 | 40 | 75 | 20 |
| Dist. to nearest settlement (km) | 6.7 | 13.3 | 29.1 | 11.3 | 28.3 | 47.6 |
| Dist. to paved road (km) | 0.01 | 11.8 | 0.01 | 0.2 | 25.3 | 1.8 |
| Agriculture (%) | 70 | 60 | 35 | 40 | 70 | 0 |
| Alien crops* | 7 | 6 | 8 | 11 | 6 | 0 |
| Pesticides use | Frequent | Occasional | Frequent | Seldom | Seldom | Seldom |
| Livestock (ranching) | Frequent | Occasional | Frequent | Seldom | Seldom | Frequent |
| Slash-and-burn method | Frequent | Occasional | Frequent | Occasional | Seldom | Occasional |

***** Angiosperm plants only.


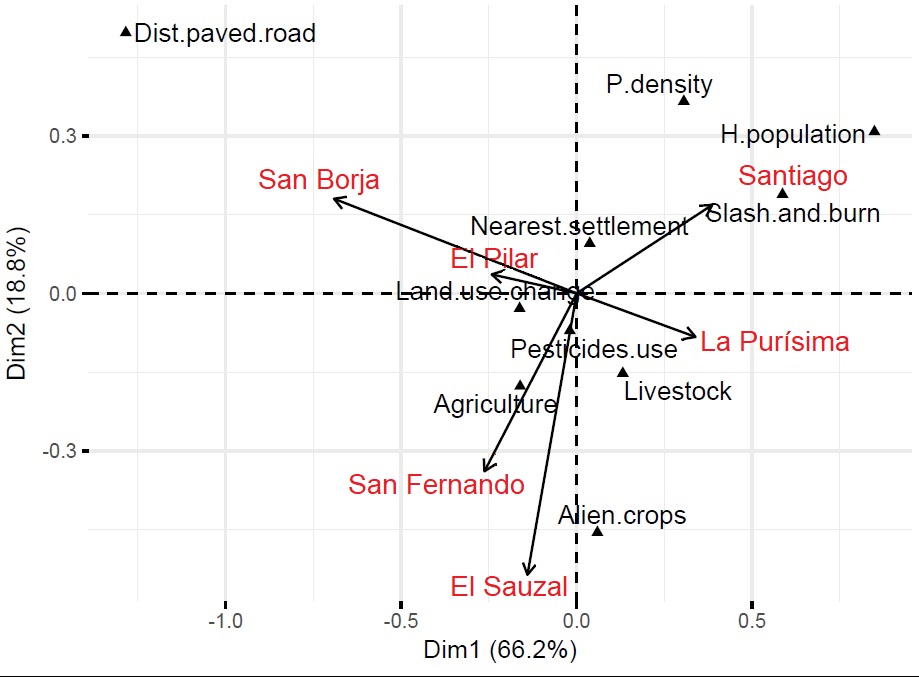


**Figure S1.** Canonic correspondence analysis showing human impacts and their effects among studied oases.


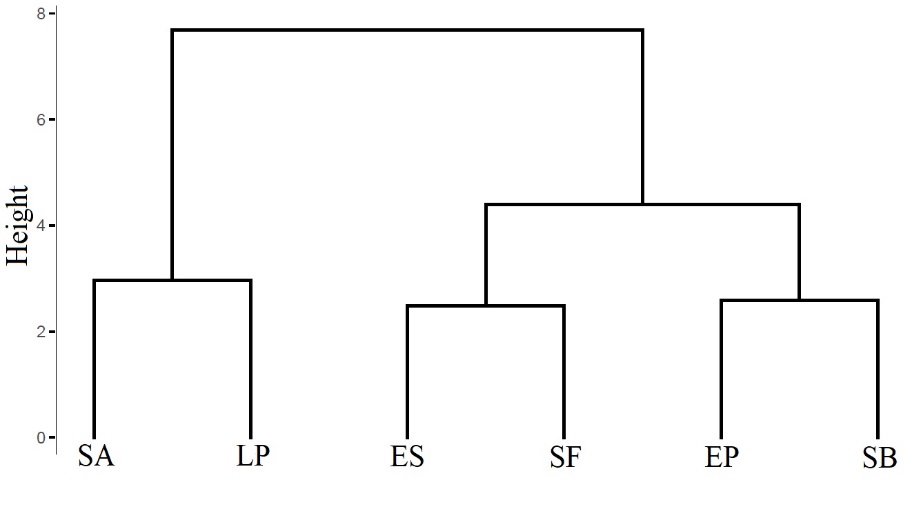


**Figure S2.** Cluster analysis in correspondence to the degree of human alteration. *K=3.* SA=Santiago, EP=El Pilar, LP= La Purísima, ES=El Sauzal, SB=San Borja, SF=San Fernando.

**Appendix S2.** We present the general steps taken to build the models. Comparisons between models for bees and wasps considered the values of lowest AICc (Second-Order Akaike Information Criterion for small samples) and the highest explained deviance, starting from a null model (Bates et al. 2015). We followed the step forward technique, incorporating those variables with biological meaning and statistical significance to the models (Zuur et al. 2009). (Table S1).

**Table S1.** Example of model selection explaining the nest abundance of bees and wasps. The △AICc value is with respect to the null model. During the selection, the minimum adequate models considered △AICc > 2 units (Zuur et al. 2009).

|  |  | Bees | | | Wasps | | |
| --- | --- | --- | --- | --- | --- | --- | --- |
| Model | Type of model | AICc | △AICc | Deviance | AICc | △AICc | Deviance |
| 1 | Null | 346 | - | 342 | 328.3 | - | 324.3 |
| Selected model | Response variable ^~^ Fixed effects + (random effect) | 328.7 | 0 | 320.7 | - | 0 | - |
| Selected model | Response variable ^~^ Fixed effects + (random effect) | - | - | - | 290 | 38.3 | 278 |

**References**

Bates, D., Maechler, M., Bolker, B., & Walker, S. (2015) Fitting Linear Mixed-Effects Models Using lme4. *Journal of Statistical Software*, 67, 1-48.

Mantorell, C., & Peters, E. M. (2005). The measurement of chronic disturbance and its effects on the threatened cactus *Mammillaria pectinifera. Biological Conservation,* 124, 199–207*.*

Zuur, A. F. Ieno, E. N., Walker, N. J., Saveliev, A. A., & Smith, G. M. (2009). *Mixed effects models and extensions in ecology with R*. New York, NY: Springer.
